# Urinary retention due to mesenteric cyst: An extremely unusual presentation of a rare complication

**DOI:** 10.4103/0971-4065.41285

**Published:** 2008-01

**Authors:** B. Ghazimoghadam, S. Rajaie

**Affiliations:** Department of Nephrology, Golestan University of Medical Sciences, Golestan province, Iran

**Keywords:** Acute urinary retention, abdominal mass, mesenteric cyst

## Abstract

Mesenteric cysts are rare intra-abdominal masses, presenting with various clinical signs and symptoms. Mesenteric cysts presenting with a sudden onset of urinary retention is extremely rare. There are no cases reported in the English literature. Here, we report a very rare case of urinary retention, due to mesenteric cyst in a 19-year-old man. The patient presented with abdominal distention with a sudden onset of urinary retention. Ultrasonography and computed tomography scan of the abdomen revealed a huge cystic mass above the bladder that was completely separated from the kidney and bladder. The cyst was removed surgically. The patient experienced no urological difficulty after the surgery. Histological examination confirmed the diagnosis of a mesenteric cyst.

Mesenteric cysts are rare, intra-abdominal lesions arising with an incidence of 1/100,000 in adults and 1/20,000 in children.^1^ Most of these cysts are benign. They can be discovered as an incidental finding during laparotomy for some other condition or can manifest as an acute life-threatening intra-abdominal catastrophe. Approximately 10% of patients with mesenteric and omental cysts present with an acute abdominal emergency.^2^

## Case Report

A 19-year-old male presented with abdominal distention and gastrointestinal obstruction occurring over a few months with a sudden onset of urinary retention.

He had been healthy until the admission and the medical and surgical history recorded was unremarkable. He was stable on physical examination, but a lower abdominal mass was palpable. No hepatosplenomegaly or clinical lymphadenopathy was detected. Urethral catheterization was performed and a volume of 1000 cc urine was drained but the mass size did not change.

laboratory investigations showed normal blood biochemical indices and gross hematuria (RBC = 20-30 in urine analysis)

Following are the findings of the imaging studies:

- Retrograde urethrography: There was no filling defect. The margins were completely smooth.
- Ultrasonography (US): A huge, cystic and echo-free mass above but separate from the bladder was visible. It was located on the right side and extended to the left to some degree. In the longitudinal axis, it was extending above the umbilicus. In the right superior part, a small echo-free area, separated with a septum from the main mass was visible. Both masses were also visible after diuresis.
- Computed tomography (CT) scan also showed a homogenous and hypodense mass with a septum and smooth margins. It was precisely above the bladder and extended to the right. The mass extended upward to the superior right margin of the bladder and was completely separated from the bladder and kidneys.

The right pelvic area was slightly distended, possibly be due to the compressive effect of the mass.

### Pathological evaluation

At laparatomy, a large pediculous mass was discovered in the abdomen. The cyst was grossly 4 × 9 × 12 cm in size. The wall thickness varied from 0.02 to 1 cm. It consisted of multiple small loculi. Histological examination of the cyst revealed a vascular connective tissue combined with the focal areas of hemorrhage and cholesterol splits and inflammatory cells infiltration. A diagnosis of a mesenteric cyst was thus confirmed.

## Discussion

Urethral obstruction is a symptom of various intra-abdominal complications such as large ovarian cysts,^3^ spinal tumors,^4^ uterine leiomyoma and retroverted gravid uterus,^5^ genital herpes simplex virus infection.^6^ In elderly patients, benign prostate hypertrophy (BPH), surgical repair for uterine prolapse and complicated appendicitis can cause acute urinary retention.^7^ One of the most uncommon etiologies of urinary retention is mesenteric cysts. Since the first report released regarding these cysts, only 820 cases have been reported.^8^ Though commonly found in the ileal and right colonic mesentery, the mesenteric cysts can be localized anywhere in the mesentery.

Mesenteric cysts are uncommon and clinically confusing lesions.^9^ The size and age of patients influence the clinical presentation.^10^–^12^ Although mesenteric cysts are most common in the 40s,^9^ they may also affect young children; moreover, they appear to have no significant gender or race predilection.^13^ Although some reports show that these cysts appear more frequently in the young and middle-aged women, some exception do exist.^14^ Children typically present with acute abdomen, while adults have more indolent symptoms.^1^,^10^,^11^

The symptoms are variable, non-pecific and include pain (82%), nausea and vomiting (45%), constipation (27%) or diarrhea (6%).^15^ An abdominal mass may be palpable in approximately 61% of patients.^1^,^15^

Desai *et al.,* from India reported a case of a giant mesenteric cyst of abdomen herniating into the scrotum.^14^ Increasing abdominal girth is an uncommon presentation of the mesenteric cysts, reported by Pantanowitz and Botero in a 39-year-old otherwise healthy man in Israel.^13^

Surgery is the mainstay of the treatment and also the only definitive diagnostic modality for simple mesenteric cysts.^16^ Aspiration a cyst alone should not be performed. Complete enucleation of the cysts is considered the procedure of choice in order to prevent its recurrence and possible malignant transformation. Successful laparoscopic resection of mesenteric cysts have been reported.^17^

In this case, the mesenteric cyst was located immediately above the urinary bladder, in the ileum site of the mesentery, from the right corner of the mesentery which compressed the bladder from the above and lateral sides. Thus, this compression caused the retention of urine in the patient.

Our hypothesis in the present case explains that the cause of urinary retention due to mesenteric cyst was the pressure that compressed the nerves of the bladder, resulting in the complication, although further complementary studies are required in order to confirm this hypothesis.
